# Phenotypic characterization of Guraghe and Jimma cattle breeds in Ethiopia: Implications for breed differentiation and *in-situ* conservation

**DOI:** 10.1371/journal.pone.0303559

**Published:** 2024-05-21

**Authors:** Amine Mustefa, Tesfalem Aseged, Hizkel Kenfo, Kebebew Hunde

**Affiliations:** 1 Ethiopian Biodiversity Institute, Animal Biodiversity Directorate, Addis Ababa, Ethiopia; 2 Ethiopian Biodiversity Institute, Hawassa Biodiversity Center, Hawassa, Ethiopia; Universidade Federal de Mato Grosso do Sul, BRAZIL

## Abstract

The aim of this study was to phenotypically characterize Guraghe and Jimma cattle breeds at the farm level and quantify the relationships between them. Eight morphometric measurements and sixteen morphological traits were recorded for a total of 313 (221 females and 92 males) randomly selected adult cattle from three purposively selected districts. Univariate and multivariate analysis procedures of Statistical Analysis Software (SAS 9.0) were used to analyze the data. Clear morphological and morphometric variations were not observed between the two cattle breeds. All of the studied cattle populations possessed straight-edged ears, a sloppy rump profile, and straight face and back profiles. Moreover, the majority of the studied cattle possess widely spaced curved horns, red-colored uniform body color patterns, and erected small humps located at the cervicothoracic position. In addition to their phenotypic similarities, multivariate analysis also failed to reveal significant differences between the two breeds. These results suggest the inseparable nature of the two cattle breeds. However, such similarities in phenotypic traits between the two cattle breeds do not necessarily indicate genetic similarities. Therefore, further genetic characterization is recommended to quantify the degree of genetic relationship between the breeds. In the meantime, it is recommended to design breed-specific in situ conservation as well as genetic improvement programs that consider cattle breeds as one. Furthermore, an inclusive and uniform breed name that can represent the two cattle populations is obtained from the country’s steering committee for indigenous animal genetic resources.

## Introduction

Cattle are among the most important livestock species in Ethiopia, with high contributions to the gross domestic product (GDP) of the country in general and to that of individual farmers [1]. Farmers keep cattle for various reasons, including meat, milk, drought power, sources of income, and sources of manure, and as cultural and social entities [2–6]. Cattle are the most populous livestock species in Ethiopia, with a reported total of 66.3 million head [1]. Similarly, this number also makes the country the country with the largest cattle population in Africa [7].

Within and among breeds, diversity is the most important public asset that can be used as an input for selection-based genetic improvement and conservation programs. In addition to the existence of some exotic breeds introduced for genetic improvement purposes, the majority of Ethiopian cattle populations are indigenous and consist of 28 registered breeds [8,9]. This makes them the most diverse species in the country. However, the actual number of cattle is expected to increase due to the presence of some studied but unregistered cattle breeds, including the Bonga, Fellata, Gamo, Hadiya, Jimma, Mahibere-Silassie composite, Maya, Qocherie, Ruthana, Sidama, and Wollo highland cattle breeds [9]. On the other hand, a few cattle breeds were registered without full phenotypic characterization studies. These cattle breeds include Adwa, Ambo, Bale, Borana, Guraghe, Hamer, Jem-Jem, Jigjiga, and Smada [9]. These gaps show the lack of exhaustiveness of the previous studies, which need to be addressed by further and inclusive phenotypic characterization studies [9].

The current study targeted two cattle breeds: the Guraghe and Jimma cattle breeds. Guraghe cattle are reported to be found around the Guraghe and Hadiya areas near the tsetse-infested valleys of the Ghibe tributaries [10,11]. Guraghe cattle are mainly red, chestnut and roan in body color. Guraghe cattle are used mainly for draught and milk production; however, for several reasons, including tsetse infestation and low pasture conditions in their production areas, they do not perform either of these functions well [10]. The other breed, Jimma cattle, is reported to be found in the Dedo, Gera, and Sigimo districts of the Jimma zone [12]. The Jimma zone is adjacent to the Guraghe zone, where the tsetse-infested Ghibe valley separates the two zones. The majority of Jimma cattle possess red and brown body colors. Jimma oxen were selected for their draught performance, large body, body color preference, and growth performance, while Jimma cows were selected for their milk yield, body color and size, and fertility [12].

The Guraghe cattle breed is one of the 28 registered cattle breeds in Ethiopia [8]; however, it has not been phenotypically characterized. The registration of this cattle breed was made solely based on the report of [10], which is the first country-wide simple assessment study. On the other hand, the Jimma cattle breed was phenotypically characterized eleven years ago [12]; however, it was not registered in the country’s indigenous animal database. Moreover, these two cattle breeds are neighbors of one another. Therefore, an inclusive phenotypic characterization study involving a representative sample from the two cattle breeds is required to update the information we have about the two cattle breeds and to contribute to the breed differentiation and registration process. Moreover, documenting Guraghe cattle as well as updating the phenotypic characteristics of Jimma cattle are important for designing breed-specific genetic improvement and conservation programs. Thus, the current study aimed to characterize the on-farm phenotype of Guraghe and Jimma cattle breeds and quantify the relationships between them.

## Materials and methods

### Ethics approval and consent of participants

The current study was approved by the Ethiopian Biodiversity Institute (EBI) from ethical and technical perspectives. The EBI is the primary institution responsible for the characterization, conservation and sustainable utilization of indigenous animal, plant and microbial genetic resources. Each participant farmer agreed to allow their animals to be measured using a centimeter-scale measuring tape. The measurements were carried out according to the [13] guidelines for phenotypic characterization of farm animal genetic resources for food and agriculture. Additionally, permission regarding access to the field sites was obtained from the agricultural offices of the three districts: the Meskan district Agriculture Office, the Abeshge district Agriculture Office, and the Dedo district Agriculture Office.

### Study areas

The study was carried out in three districts, i.e., Meskan, Abeshge, and Dedo. The Meskan and Abeshge districts were selected from the Guraghe zone of the central Ethiopian region, while Dedo district was selected from the Jimma zone of the Oromia region. The agroecology- and weather-related parameters of the sampled districts are presented in Table 1. The sizes of the cattle populations in the Guraghe and Jimma zones were estimated to be 1,068,510 and 2,629,417, respectively [1].

**Table 1:** Information on the agroecology and weather conditions of the selected districts.

Parameters	Districts		
	Meskan	Abeshge	Dedo
Altitude of the district (m.a.s.l.)	1840–3200	1400–2900	880–3058
Altitude of the sampled locations (m.a.s.l.)	2131–2488	1737–1891	1717–2832
Temperature (°C)	10.3–26.0	21.25	11–29
Rainfall (mm)	900–1400	801–1400	1300–1700
Area (km^2^)	446.7	559.0	1,516
Human population	218,370	79,901	407,175
Ethnicity	Guraghe	Guraghe	Oromo

Key: m.a.s.l. = meters above sea level,°C = degrees Celsius, mm = millimeters, km^2^ = square kilometers.

Sources: [14–18].

### Site and animal selection

Representative samples of Guraghe and Jimma cattle breeds were selected from their respective breeding tracts. Information regarding their breeding tract and distribution areas was obtained using secondary information. The Guraghe zone was reported to be the breeding tract of Guraghe cattle, but its distribution can reach the neighboring Silte and Hadiya zones [9,10]. Therefore, two districts (Meskan and Abeshge) were randomly selected from the Guraghe zone to represent the indigenous Guraghe cattle. On the other hand, Jimma cattle are reported to be found in the Dedo, Gera, and Sigimo districts of the Jimma zone [12]. Therefore, one district (Dedo) was selected randomly from the three districts. Two sampling sites (***Kebeles***) were randomly selected from each district. Households in which cattle were reared were randomly selected from each sampling site (***kebele***). Two unrelated adult cattle aged four years and older were randomly selected from each household. The selected animals were controlled carefully by their owners and trained laborers. Aggressive animals that could not properly stand on flat ground were not measured.

### Data collection

Data on morphometric (quantitative linear body measurements) and morphological (qualitative characteristics) traits were collected based on the data collection procedures of the [13] guidelines. Data collection was performed in the morning to avoid errors regarding feeding and watering. Four researchers participated in the data collection procedure: two handled the quantitative data, while the remaining two handled the qualitative data decision making and recording. To reduce bias, morphometric data recording was performed by the same researcher throughout the study. Animals were measured using a centimeter-unit textile measurement tape. A total of 313 cattle (221 females and 92 males) were subjected to eight morphometric measurements (Table 2) and sixteen morphological/qualitative traits.

**Table 2:** Explanations of the linear body measurements.

No.	Morphometric traits	Definitions
1	Body length	Distance from shoulder point to pin bone
2	Heart girth	Chest circumference right behind its front two legs
3	Height at withers	Distance from ground to withers of the front foot
4	Pelvic width	Distance between the two ends of the pelvic bone
5	Muzzle circumference	Perimeter of the mouth
6	Ear length	Distance from the root to the tip of the back side of the ear
7	Horn length	Outer side distance between root and tip of the horn
8	Cannon bone length	Distance between the fetlock joint (ankle) and the knee

Source: [13].

### Data analysis

The Microsoft Office Excel worksheet was used to enter and manage the data, and the overall data analysis was carried out using the Statistical Analysis System (SAS) software 9.0 [19].

#### Univariate analysis

The UNIVARIATE procedure for the data normality test, the frequency procedure for morphological (qualitative) data analysis, and the general linear model (GLM) procedure for morphometric (quantitative) data analysis were used. The following analysis model was used to analyze the morphological data. Y_ij_ = μ + S_i_ + B_j_ + e_ij,_ where Y_ij_ is an observation, μ is the overall mean, S_i_ is the fixed effect of sex (i = male, female), B_j_ is the fixed effect of breed (j = Guraghe and Jimma) and e_ij_ is the random error. The quantitative data were analyzed separately for each sex by fitting the breed as a class variable. The means (LSMs) of the quantitative data were separated using the adjusted Tukey–Kramer test.

#### Multivariate analysis

Stepwise discriminant analysis to detect morphometric traits that better discriminate the cattle breeds, discriminant analysis to allocate individuals to known breeds and assess the possibility of misclassifications, and canonical discriminant analysis to determine maximal separations between breeds were used. Graphic interpretation of breed differences was plotted using the scored canonical variables. The pairwise Mahalanobis distances between breeds were computed as:

*D*^2^(*i*|*j*) = (*x*_*i*_−*x*_*j*_)′*cov*^−1^(*x*_*i*_−*x*_*j*_). where *D*^2^(*i*|*j*) is the distance between breeds *i* and *j*, *cov*^−1^ is the inverse of the covariance matrix of measured variables, and *x*_*i*_ and *x*_*j*_ are the means of variables in the *i*^th^ and *j*^th^ breeds, respectively.

## Results

### Qualitative characteristics

**Fig 1:**
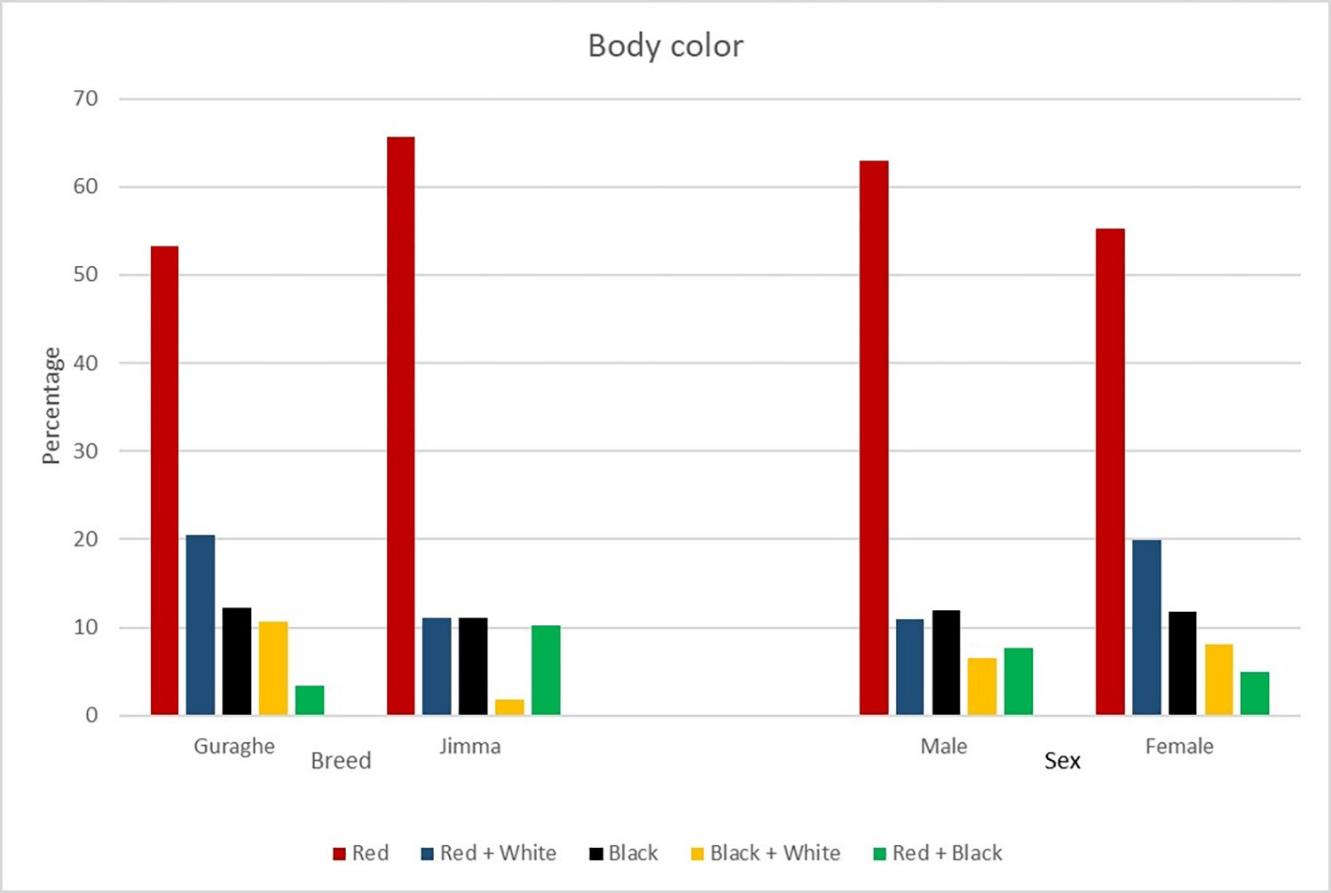
Effect of breed on body color (chi-square value 18.53, p = 0.001) and effect of sex on body color (chi-square value 4.78, p = 0.3108).

The body colors of the two studied breeds and sexes are presented in Fig 1. Body color was not significantly affected by the sex of the animals, while it was affected by their breed. In addition to the dominance of red body color in the majority of the cattle populations, red + white cattle were more frequently observed in Guraghe than in Jimma.

The effects of breed and sex on the qualitative characteristics of the Guraghe and Jimma cattle breeds are presented in Table 3, along with the respective *chi-*square values and levels of significance. Thirteen out of the fifteen qualitative characteristics were not significantly affected by the cattle breed, while sex affected seven traits. Accordingly, all of the studied cattle populations possessed straight edged ears, a sloppy rump profile, and straight face and back profiles. Moreover, the majority of the studied cattle possess widely spaced curved horns, red-colored uniform body color patterns, and erected small humps located at the cervicothoracic position. Furthermore, they possess medium to long tails, medium dewlap widths and perpetual sheaths. Most of the female cattle populations did not possess naval flaps, while a considerable number of cattle exhibited small to medium naval flap widths. In terms of sex, laterally oriented straight horns were more frequently observed in males than in females. The majority of females possess erected humps, while some males possess dropping humps. Males also had larger humps located at the thoracic position, while females possessed small humps located at the cervicothoracic position.

**Table 3:** Percentages of qualitative characteristics of cattle populations by sex and breed.

Qualitative traits		Cattle Breed				Sex			
		Guraghe	Jimma	*X*^*2*^ value	P	Oxen	Cows	*X*^*2*^ value	P
N		205	108			92	221		
Horn spacing	Narrow	24.4	23.2	0.06	NS	31.5	20.8	4.1	*
	Wide	75.6	76.8			68.5	79.2		
Horn shape	Straight	45.4	44.4	0.02	NS	72.8	33.5	40.6	***
	Curved	54.6	55.6			27.2	66.5		
Horn orientation	Lateral	36.6	36.1	1.8	NS	66.3	24.0	51.6	***
	Upright	34.6	32.4			21.7	38.9		
	Forward	21.5	26.9			8.7	29.4		
	Dropping	7.3	4.6			3.3	7.7		
Color pattern	Uniform	65.8	76.9	9.3	*	71.7	68.8	0.4	NS
	Spotty	5.4	2.8			4.4	4.5		
	Pied	15.1	4.6			9.8	12.2		
	Shaded	13.7	15.7			14.1	14.5		
Hump shape	Erect	94.2	95.4	0.21	NS	81.5	100	43.2	***
	Dropping	5.8	4.6			18.5	0		
Hump size	Small	56.1	69.4	5.4	NS	6.5	83.3	196.0	***
	Medium	25.4	16.7			37.0	16.3		
	Large	15.5	13.9			56.5	0.4		
Hump position	Thoracic	38.5	30.6	2.0	NS	93.5	11.8	188.8	***
	Cervico thoracic	61.5	69.4			6.5	88.2		
Tail length	Short	12.2	12.0	3.6	NS	13.1	11.7	3.0	NS
	Medium	50.2	39.8			39.1	49.8		
	Long	37.6	48.2			47.8	38.5		
Dewlap width	Small	13.2	20.4	3.2	NS	2.2	21.3	65.3	***
	Medium	72.7	63.9			59.8	73.7		
	Large	14.1	15.7			38.0	5.0		
Naval flap width	Absent	51.7	61.6	4.2	NS	-	54.8	NA	NA
	Small	34.2	30.1			-	33.0		
	Medium	12.8	5.5			-	10.4		
	Large	1.3	2.8			-	1.8		
Perpetual sheath	Absent	1.7	0	11.5	**	1.1	-	NA	NA
	Small	24.2	42.9			30.4	-		
	Medium	72.4	42.9			62.0	-		
	Large	1.7	14.2			6.5	-		
Ear shape	Straight edged	100	100	NA	NS	100	100	NA	NS
Face profile	Straight	100	100	NA	NS	100	100	NA	NS
Back profile	Straight	100	100	NA	NS	100	100	NA	NS
Rump profile	Sloppy	100	100	NA	NS	100	100	NA	NS

Key: *X*^*2*^ = chi-square test, P = probability

* = p < 0.05

** = p < 0.01

*** = p < 0.0001, NS = not significant, NA = not available.

### Morphometric traits

The means (least squares), standard errors and pairwise comparisons showing the effects of breed on the morphometric traits of the studied male and female cattle populations are presented in Table 4. The effect of breed on the morphometric measurements was found to be sex dependent. The measurements of the males were significantly affected by breed differences compared with the measurements of the female cattle populations. Within the female cattle population, Jimma cows had a significantly longer body than did Guraghe cows, while Guraghe cows had significantly greater heart girth and height at withers than did Jimma cows. However, the other body measurements did not show significant differences between the female cattle populations. On the other hand, within the male cattle populations, significantly greater heart girth, height at wither, pelvic width, muzzle circumference, and horn and ear length measurements were observed for the Guraghe oxen than for the Jimma oxen, while the two breeds had comparable body and ear length measurements.

**Table 4:** The effect of breed on the morphometric measurements of cattle by sex.

Traits	Cows			Oxen		
	Guraghe	Jimma	*p*	Guraghe	Jimma	*p*
N	148	73		57	35	
BL	99.6±0.45	101.8±0.63	**	107.6±1.10	106.0±1.41	NS
HG	130.6±0.51	124.8±0.72	***	143.9±1.28	135.1±1.63	***
HW	105.6±0.34	104.4±0.48	*	112.5±0.66	107.2±0.85	***
PW	32.6±0.16	32.6±0.23	NS	34.2±0.42	32.5±0.53	*
MC	36.2±0.15	36.0±0.21	NS	40.2±0.38	38.9±0.48	*
EL	16.5±0.11	16.3±0.16	NS	17.2±0.20	16.9±0.26	NS
HL	18.7±0.53	19.9±0.76	NS	19.8±0.83	14.8±1.05	**
CBL	18.6±0.08	18.5±0.11	NS	19.3±0.15	18.6±0.19	**

Key: N = number of animals sampled, BL = body length, HG = heart girth, HW = height at the withers, PW = pelvic width, MC = muzzle circumference, EL = ear length, HL = Horn length, CBL = Cannon bone length

* = p < 0.05

** = p < 0.01

*** = p < 0.0001, NS: not significant.

The means (least squares), standard errors and pairwise comparisons showing the effect of location on the morphometric traits of Guraghe cattle are presented in Table 5. Body measurements were significantly greater for the Guraghe cattle sampled from the Abeshge district than for those sampled from the Meskan district, except for the measurements of cannonbone length. On the other hand, almost half of the morphometric measurements of Guraghe cattle were not affected by location differences.

**Table 5:** The effect of location on the morphometric measurements of Guraghe cattle by sex.

Traits	Guraghe cows			Guraghe oxen		
	Meskan	Abeshge	*p*	Meskan	Abeshge	*p*
N	68	80		27	30	
BL	97.8±0.62	101.1±0.58	**	102.4±1.24	112.3±1.17	***
HG	129.4±0.78	131.7±0.72	*	138.4±1.65	148.9±1.57	***
HW	104.0±0.48	107.0±0.44	***	110.8±0.94	114.1±0.89	*
PW	31.6±0.24	33.4±0.22	***	32.1±0.56	36.2±0.52	***
MC	36.5±0.23	35.9±0.21	NS	39.5±0.50	40.8±0.47	NS
EL	16.4±0.16	16.5±0.15	NS	17.0±0.30	17.3±0.29	NS
HL	18.0±0.79	19.3±0.73	NS	19.3±1.30	20.3±1.23	NS
CBL	18.9±0.13	18.3±0.12	**	19.5±0.23	19.0±0.22	NS

Key: N = number of animals sampled, BL = body length, HG = heart girth, HW = height at the withers, PW = pelvic width, MC = muzzle circumference, EL = ear length, HL = Horn length, CBL = Cannon bone length

* = p < 0.05

** = p < 0.01

*** = p < 0.0001, NS: not significant.

### Multivariate analysis

#### Stepwise discriminant analysis

Five out of the eight morphometric traits were used for discriminating the males, while only two of them were used for discriminating the females (Table 6). Heart girth and body length were among the measurements used to discriminate between the male and female cattle populations. However, overall low partial R-square and F values were observed.

**Table 6:** Order of traits used in discriminating the cattle populations from different breeds.

Sex	Step	Variables entered	Partial R-Square	F value	Pr > F	Wilks’ Lambda	Pr < Lambda
Cows	1	Heart girth	0.1650	43.28	<0.0001	0.8349	<0.0001
	2	Body length	0.1589	41.17	<0.0001	0.7023	<0.0001
Oxen	1	Height at withers	0.2150	24.65	<0.0001	0.7849	<0.0001
	2	Horn length	0.0716	6.86	0.0104	0.7288	<0.0001
	3	Body length	0.0482	4.45	0.0376	0.6936	<0.0001
	4	Heart girth	0.0412	3.74	0.0563	0.6650	<0.0001
	5	Muzzle circumference	0.0242	2.13	0.1478	0.6489	<0.0001

#### Discriminant analysis

The results of the discriminant function analysis showed moderate classification (74.5% for the females and 73.3% for the males) of individual animals into their corresponding breed (Table 7). The greatest classification into their respective breeds was observed in Jimma oxen, while the lowest classification was observed in Guraghe oxen.

**Table 7:** Numbers and percentages of observations classified into breeds.

Sex	From breed	Guraghe	Jimma	Total
Cows	Guraghe	**109 (73.65)**	39 (26.35)	148 (100)
	Jimma	18 (24.66)	**55 (75.34)**	73 (100)
	Error rate	0.2635	0.0.2466	**0.2550**
Oxen	Guraghe	**38 (66.67)**	19 (33.33)	57 (100)
	Jimma	7 (20)	**28 (80)**	35 (100)
	Error rate	0.3333	0.2	**0.2667**

#### Canonical discriminant analysis

The eigenvalues and canonical correlation outputs for both male and female cattle populations are shown in Table 8. In line with the low partial R-square and F value outputs in Table 7, the eigenvalues were also small enough to discriminate between the two cattle breeds of both sexes. Similarly, the canonical correlations that were used to construct canonical variate 1 (Can 1) from the morphometric measurements for both sexes were also low.

**Table 8:** Multivariate statistics outputs of the canonical structure.

Multivariate Statistics	Females	Males
Canonical correlation	0.5550	0.5930
Eigen value	**0.4452**	**0.5424**

The pairwise squared Mahalanobis distances are presented in Table 9. According to the results, the oxen of the two breeds were more distantly related than those of the cows. However, both distances were short enough to declare a significant distance between the breeds. The overall multivariate analysis results revealed small and nonsignificant differences between the Guraghe and Jimma cattle breeds.

**Table 9:** Pairwise squared distances between breeds: cows above diagonal, oxen below diagonal.

From District	Guraghe	Jimma
Guraghe	**0**	1.99
Jimma	2.25	**0**

**Fig 2:**
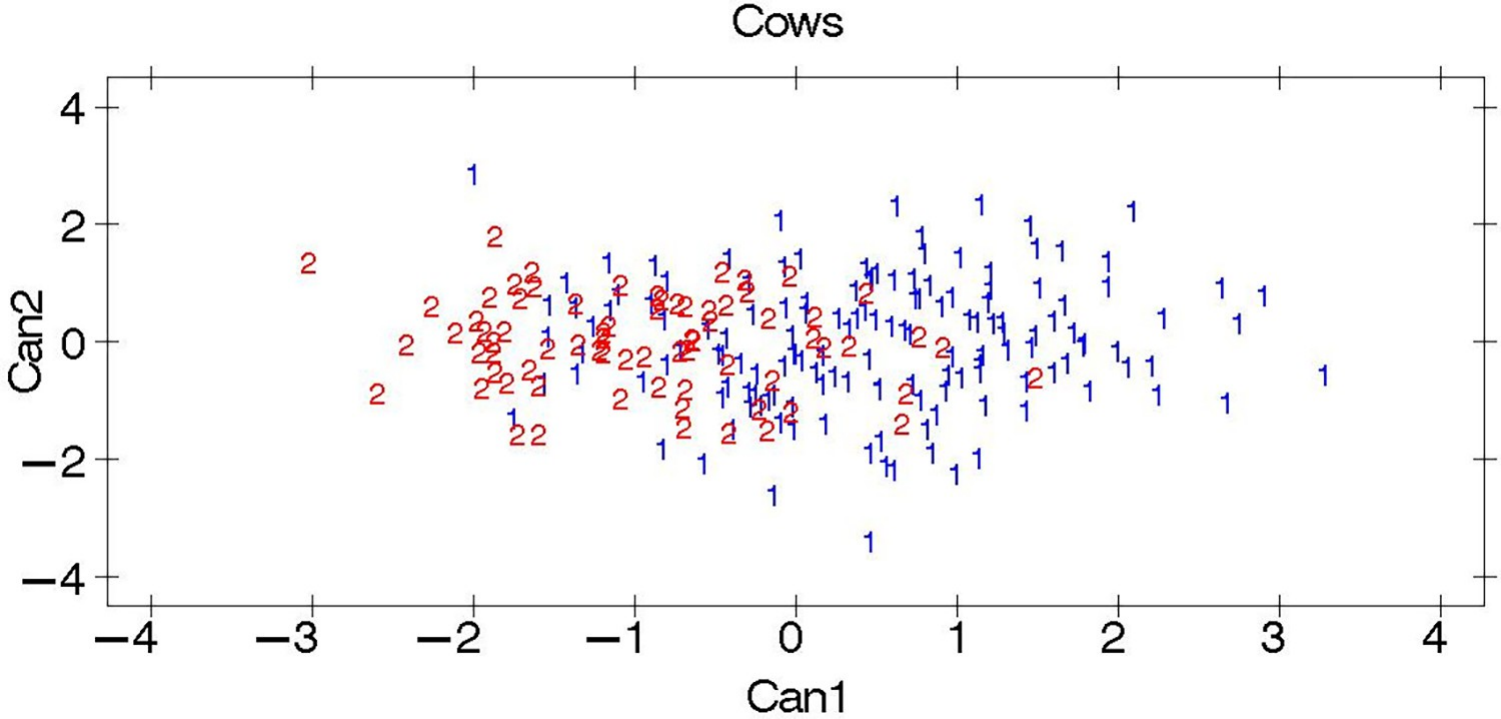
Plots of canonical discriminant analysis for cows. The breeds are indicated by the numbers 1: Guraghe and 2: Jimma.

**Fig 3:**
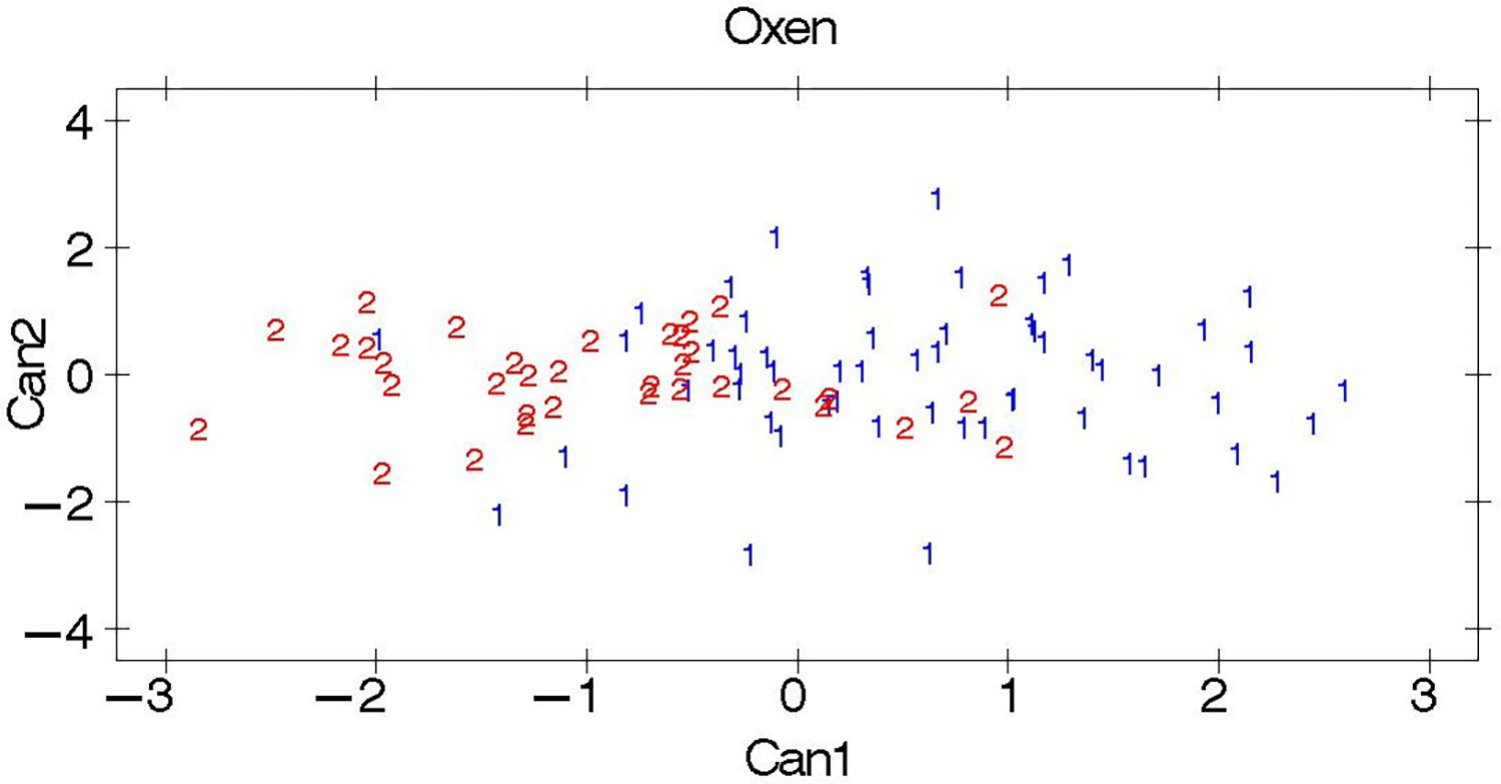
Plots of canonical discriminant analysis for oxen. The breeds are indicated by the numbers 1: Guraghe and 2: Jimma.

Plots of the first two canonical variables used to discriminate the cattle breeds are presented in Figs 2 and 3. In line with the results of the Mahalanobis distances, the studied Guraghe and Jimma cattle breeds were inseparable and categorized into the same group, while relative separation was observed between the males.

## Discussions

### Qualitative characteristics

Due to their easily observable nature, qualitative characteristics can help us simply distinguish breeds. Body color and body color patterns are among the easily observable qualitative traits that can be used to differentiate breeds. However, these traits failed to differentiate Guraghe and Jimma cattle breeds, as the majority of both cattle breeds possess a uniformly patterned red body color. In addition to similarities in other morphometric and morphological traits, similarities in body color and body color patterns among breeds may suggest genetic similarity [20,21] for Ogaden cattle. Diverse body colors might be observed within a breed [21] of Harar cattle; however, the current body color and body color pattern similarities between the Guraghe and Jimma cattle breeds show phenotypic similarities, which in turn hints at the presence of genetic similarities among them. This finding needs to be confirmed by further molecular characterization.

The red-dominated body color of Guraghe cattle found in this study is in line with the reports of [10], which showed the dominance of red followed by chestnut and roan body colors in Guraghe cattle. Similarly, the red-dominated body color of Jimma cattle found in this study is also in line with the results of [12], which showed the dominance of red followed by brown body color in Jimma cattle. The dominantly observed red body color might also be associated with the farmers’ preferences and selection criteria, as red body color was strongly favorable in the studied areas. [12] also reported body color as the farmer selection criterion following draught power and body size.

In addition to similarities in body color, similarities in other qualitative characteristics were also observed between the Guraghe and Jimma cattle breeds. The uniformities of the horn, hump, tail, dewlap, naval flap, and perpetual sheath characteristics in addition to their perfect matches in ear shape, face, back, and rump profiles between the Guraghe and Jimma cattle breeds contradict their classifications as different breeds. The slight differences observed might be due to within-breed differences. Such differences within the same breed sampled from different locations were also reported by [22] in Mursi cattle, [23] in Raya cattle, and [21] in Harar cattle.

### Morphometric traits

The morphometric measurement results, in addition to the qualitative characteristics, can produce reliable information for quantifying the degree of relationships between the breeds. Within the female cattle populations, the effect of breed was not significant for most of the morphometric traits, which supports the observed qualitative resemblances between the two cattle breeds. On the other hand, most of the quantitative traits of the male cattle populations were significantly affected by their breed, revealing the greater hugeness of Guraghe oxen than of Jimma oxen. The observed sex-dependent effect of breed on morphometric traits might be due to differences in the production system between the two breeds, as farmers in the Guraghe area use special management and strong selection criteria for their oxen rather than for their cows. However, when we considered the results for both sexes, clear differences that can lead to breed differentiation were not observed. As mentioned in the qualitative section, the differences observed between the two cattle breeds can be considered within-breed variation.

Similarly, within-breed variation was also observed within the Guraghe cattle, where the sampling location significantly affected the morphometric measurements; i.e., the cattle populations from the Abeshge district had relatively greater values than did the cattle populations from the Meskan district. Such within-breed variations are among the most important factors in selection-based genetic improvement programs as well as conservation schemes. The authors of [22] in Mursi cattle, [23] in Raya cattle, and [21] in Harar cattle also reported the existence of morphometric variations among different sampling locations within the same breed.

According to [10], Guraghe cattle were also reported to be distributed in the Hadiya zone, which is one of the neighboring zones to the Guraghe zone. Phenotypic characterization was performed by [24] on indigenous cattle found in the Hadiya zone, and the values of morphometric traits were reported. Accordingly, the majority of the morphometric measurement results of the current study were comparable with the results of [24]. This proves the similarities of the cattle populations found in the two neighboring zones: the Guraghe and Hadiya zones.

The Guraghe and Jimma cattle breeds were found to be smaller in size than most of the Ethiopian indigenous cattle breeds. Their morphometric measurements were lower than those of Afar cattle [25], Begait cattle [26], Begaria cattle [6], Fogera cattle [27], Gofa cattle [5], Gojjam Highland cattle [28], Harar cattle [21], Kereyu cattle [29], Mursi cattle [22], Nuer cattle [30], Ogaden cattle [21] and Raya cattle [23]. On the other hand, the morphometric measurements of the Abergelle and Irob cattle breeds [31] were reportedly lower than those of the Guraghe and Jimma cattle breeds. Comparable morphometric traits were also reported in Arado cattle [3] and Horro cattle [32].

The males were larger and larger than the females for most of the morphometric traits of both breeds. This dominance of the males was in line with Rensch’s rule [33], which stated that females of a particular species are usually smaller than males. Moreover, such differences might be due to the secretion of testosterone hormones by males, which leads to the development of skeletal and muscle mass [34]. The effect of the endocrine system was also significant for differentiating the two sexes, as the effect of estrogen on growth was limited in females [34,35]. Comparable results showing the dominance of males over their female counterparts were also reported by [21] for the Harar and Ogaden cattle breeds, [23] for the Raya cattle breed, and [22] for the Mursi cattle breed.

### Multivariate analysis

High R-square and F values of the morphometric traits were also required to determine the significance of the discrimination results. On the other hand, low error rates were required to indicate the presence of separate breeds. However, the stepwise analysis (Table 6) revealed low R-square and F values, which showed the weak potential of the morphometric traits to discriminate the cattle populations. Moreover, in support of this, high error rates (Table 7) were observed, showing greater similarities between the studied cattle populations. This results in a lower chance of the breeds being categorized into separate clusters. On the other hand, the lower the error rate is, the lower the similarities shared among the breeds, which indicates the uniqueness of each breed. An error rate of 1% was reported in classifying the phenotypically unrelated Harar and Ogaden cattle breeds [21].

An eigenvalue higher than 1 is needed to declare discrimination between the breeds. If the value is lower than 1, the discrimination of the studied animals into separate breeds is insignificant. Accordingly, the lowest Eigenvalue results observed for both sexes (Table 8) did not support the categorization of the studied cattle populations into different clusters, which disproves the presence of two separate breeds. Similarly, high Mahalanobis distance values between the two cattle breeds are needed to classify them into separate clusters. However, the Mahalanobis distance results (Table 9) of the current study were low, while the males showed relatively greater distances. The relatively greater Mahalanobis distances between the males might be due to the small number of oxen sampled. The accuracy of the analysis increased as the number of sampled animals increased. Due to the low Eigenvalue (<1) and short Mahalanobis distance outputs of the multivariate analysis, the studied cattle breeds were found to be phenotypically inseparable. However, such phenotypic similarities do not necessarily indicate genetic similarities between the breeds [36]. Other studies [23] on Raya cattle and [21] on Harar and Ogaden cattle also reported comparable results.

## Conclusion

Morphometric measurements and morphological characteristics were used to phenotypically characterize Guraghe and Jimma cattle breeds at the farm level as well as to quantify their phenotypic relationships. Similarities in most of the morphological traits were observed between the two cattle breeds. Accordingly, all cattle of both breeds possess straight edged ears, a sloppy rump profile, and straight face and back profiles. Moreover, most of them possess widely spaced curved horns, red-colored uniform body color patterns, and erected small humps located at the cervicothoracic position. Furthermore, they possess medium to long tails, medium dewlap widths and perpetual sheaths. Similarly, clear differences in morphometric traits that can lead to breed differentiation were not observed. In addition to the presence of major similarities in morphological and morphometric traits between the two breeds, multivariate analysis also revealed no significant differences between the Guraghe and Jimma cattle breeds. These results suggest the inseparable nature of the two cattle breeds. However, such similarities in phenotypic traits between the two cattle breeds do not necessarily indicate genetic similarities. Therefore, further genetic characterization is recommended to quantify the degree of genetic relationship between these breeds. In the meantime, it is recommended not to consider the studied cattle populations as separate breeds. Moreover, breed-specific in situ conservation and genetic improvement programs need to be designed considering the cattle breeds as one. Furthermore, an inclusive and uniform breed name that is able to represent the two cattle populations is obtained from the country’s steering committee for indigenous animal genetic resources.

## References

[1] CSA. Agricultural Sample Survey 2021/22 (2014 E.C). Federal Democratic Republic of Ethiopia Central Statistical Agency. Report on Livestock and Livestock Characteristics Statistical Bulletin No. 594, Volume II. Addis Ababa Ethiopia. 2022; 219pp https://www.statsethiopia.gov.et/our-survey-reports/.

[2] Zerabruk M, Vangen O. The Abergelle and Irob cattle breeds of North Ethiopia: description and on-farm characterization. Animal Genetic Resources Information Bulletin. 2005; 36:7–20. doi: 10.1017/S101423390000184X

[3] Genzebu D, Hailemariam M, Belihu K. Morphometric characteristics and livestock keeper perceptions of “Arado” cattle breed in Northern Tigray, Ethiopia. Livestock Research for Rural Development. 2012; 24(6) https://www.lrrd.org/lrrd24/1/hail24006.htm.

[4] Yimamu C. In situ phenotypic characterization and production system study of Arsi cattle type in Arsi highland of Oromia Region, Ethiopia. MSc thesis, Haramaya University, Haramaya, Ethiopia. 2014.

[5] Kebede H, Jimma A, Getiso A, Zelke B. Characterization of Gofa cattle population, production system, production and reproduction performance in Southern Ethiopia. Journal of Fisheries and Livestock Production, 2017; 5(3):237. doi: 10.4172/2332-2608.1000237

[6] Getachew F, Assefa A, Getachew T, Abegaz SK, Hailu A, Mesganaw M, et al. M. On-Farm phenotypic characterization of Begaria cattle population and their production system in Guba district, North Western Ethiopia. Ethiopian Journal of Animal Production. 2020; 20(1):1–17. https://www.researchgate.net/publication/349625372_On-Farm_Phenotypic_Characterization_of_Begaria_Cattle_Population_and_Their_Production_System_in_Guba_District_North_Western_Ethiopia.

[7] Statista. Africa: countries with largest cattle population 2024. https://www.statista.com/statistics/1290046/cattle-population-in-africa-by-country/.

[8] EBI. Ethiopian National Strategy and Plan of Action for conservation and utilization of Animal Genetic Resources. 2016.

[9] Mustefa A. Implication of phenotypic and molecular characterization to breed differentiation of Ethiopian cattle. A review. Ecological Genetics and Genomics. 2023; 29:100208. DOI: https://doi.org/10.1016/j.egg.2023.100208.

[10] Rege JEO, Tawa CL. The state of African cattle genetic resources II. Geographical distribution, characteristics and uses of present-day breeds and strains. Animal Genetic Resources Information. 1999; 26:1–25. 10.1017/S1014233900001152.

[11] Assefa A, Hailu A. Ethiopian indigenous cattle breed’s diversity, distribution, purpose of keeping, and their potential threats. Journal of Biological Innovation. 2018; 7(5):770–789. https://www.jbino.com/docs/Issue05_10_2018.pdf.

[12] Sheriff O, Belay B, Haile A. On-farm Phenotypic Characterization of Indigenous Cattle Types in Jimma Zone, Southwestern Ethiopia. Ethiopian Journal of Animal Production. 2013; 13(1):1–16. https://www.researchgate.net/publication/337387402_On-farm_Phenotypic_Characterization_of_Indigenous_Cattle_Types_in_Jimma_Zone_Southwestern_Ethiopia.

[13] FAO. Phenotypic characterization of animal genetic resources. FAO Animal Production and Health Guidelines number 11, 2012. www.fao.org/docrep/015/i2686e/i2686e00.pdf.

[14] Bekele T, Yaecob T, Fikre T. Demonstration and Evaluation of Soil Drainage Technology for Haricot Bean Productivity in Waterlogged Vertisol Areas of Abeshge District, Gurage Zone, Southern Ethiopia. Engineering and Applied Sciences. 2022; 7(4):46–50. https://sciencepublishinggroup.com/article/10.11648/j.eas.20220704.11.

[15] City population. Ethiopia Population Statistics, Charts, Map and Location. 2022; Available from: https://www.citypopulation.de/en/ethiopia/admin.

[16] Ebro A, Abebe G, Oda E. Livestock feed resources in Southern Ethiopia: The case of Meskan district. Livestock Research for Rural Development. 2017; 29(214) https://www.lrrd.org/lrrd29/11/abul29214.html.

[17] Kassa M, Kifle T. Soil Test Based Fertilizer Calibration for Common Bean (Phaseolus vulgaris L.) Varieties of the Southern Ethiopia. Applied and Environmental Soil Science. 2023; Article ID 9102273. url: 10.1155/2023/9102273.

[18] Wudad A, Naser S, Lameso L. The Impact of Improved Road Networks on Marketing of Vegetables and Households’ Income in Dedo District, Oromia Regional State, Ethiopia. Heliyon. 2021; 7: e08173 doi: 10.1016/j.heliyon.2021.e08173 34703930 PMC8524753

[19] SAS. Statistical Analysis System. Version 9.0 for windows. SAS Institute Inc., Cary NC, USA. 2002. https://www.sas.com/enus/home.html.

[20] Getachew F., Abegaz S., Misganaw M., and Fekansa T. (2014). On-farm phenotypic characterization of Ogaden cattle populations of Jigjiga zone, south eastern Ethiopia. Ethiopian Journal of Animal Production 14, 66–83. url: https://www.researchgate.net/publication/325011260_On-farm_phenotypic_characterization_of_Ogaden_cattle_populations_of_Jigjiga_zone_southeastern_Ethiopia.

[21] Mustefa A, Aseged T, Sinkie S, Getachew F, Fekensa T, Misganaw M, et al. Phenotypic diversity between and within Harar and Ogaden cattle breeds in eastern Ethiopia: The first step for conservation. Genetic Resources. 2023; 4(7):56–67. 10.46265/genresj.IXPJ9541.

[22] Terefe E, Dessie T, Haile A, Mulatu W, Mwai O. On-farm phenotypic characterization of Mursi cattle in its production environment in South Omo Zone, Southwest Ethiopia. Animal Genetic Resources. 2015; 57:15–24. 10.1017/S2078633615000132.

[23] Mustefa A, Belayhun T, Melak A, Hayelom M, Tadesse D, Hailu A, et al. Phenotypic characterization of Raya cattle in northern Ethiopia. Tropical Animal Health and Production. 2021; 53:48. 10.1007/s11250-020-02486-1. 33242126

[24] Lombebo WA, Zeleke NA. On Farm phenotypic characterization of local cattle populations in Hadiya Zone, Southern Region, Ethiopia. Journal of Advances in Dairy Research. 2018; 6:218. 10.4172/2329-888X.1000218.

[25] Tadesse D, Ayalew W, Hegde BP. On-farm Phenotypic Characterization of Cattle Genetic Resources in South and North Wollo Zones of Amhara Region, North Eastern Ethiopia. Ethiopian Journal of Animal Production. 2008; 8(1):22–38. https://www.researchgate.net/publication/284724322_On-farm_Phenotypic_Characterization_of_Cattle_Genetic_Resources_in_South_and_North_Wollo_Zones_of_Amhara_Region_North_Eastern_Ethiopia.

[26] Ftiwi M. Production system and phenotypic characterization of Begait cattle, and effects of supplementation with concentrate feeds on milk yield and composition of Begait cows in Humera ranch, Western Tigray, Ethiopia. PhD Dissertation, Addis Ababa University. 2015. http://etd.aau.edu.et/bitstream/handle/123456789/4989/Mulugeta%20Ftiwi.pdf?sequence=1.

[27] Girma E, Alemayehu K, Abegaze S, Kebede D. Phenotypic characterization, population structure, breeding management and recommend breeding strategy for Fogera cattle (Bos indicus) in Northwestern Amhara, Ethiopia. Animal Genetic Resources. 2016; 58:13–29. 10.1017/S2078633616000035.

[28] Getachew FK, Ayalew W. On-farm phenotypic characterization of indigenous cattle populations of Awi, East and West Gojjam Zones of Amhara Region, Ethiopia. Research Journal of Agriculture and Environmental Management. 2014; 3(4):227–237. https://www.researchgate.net/publication/263031309_On-farm_phenotypic_characterization_of_indigenous_cattle_populations_of_Awi_East_and_West_Gojjam_Zones_of_Amhara_Region_Ethiopia.

[29] Nigatu YM, Tadesse Y. Morphological Variations of Arsi, Kereyu and their Crossbred Cattle under current climate change in mid Rift Valley of Oromia, Ethiopia. Academic Research Journal of Agricultural Science and Research. 2020; 8(6):630–648. 10.14662/ARJASR2020.440.

[30] Minuye N, Abebe G, Dessie T. On-farm description and status of Nuer (Abigar) cattle breed in Gambella Regional State, Ethiopia. International Journal of Biodiversity and Conservation. 2018; 10(6):292–302. 10.5897/IJBC2017.1168.

[31] Zegeye T, Belay G, Hanotte O. Multivariate characterization of phenotypic traits of five native cattle populations from Tigray, Northern Ethiopia. Tropical Animal Health and Production. 2021; 53:212. doi: 10.1007/s11250-021-02652-z 33738653

[32] Bekele DT. On farm phenotypic characterization of indigenous cattle and their production systems in Bako Tibe and Gobu Sayo districts of Oromia Region, Ethiopia. MSc thesis, Haramaya University, Haramaya, Ethiopia. 2015. http://ir.haramaya.edu.et/hru/bitstream/handle/123456789/3098/Dereje%20Bekele.pdf?sequence=1.

[33] Rensch B. Die Abhangigkeit der relative sexual differenz von der korpergrosse. Bonner Zoologische Beitrage, 1950; 1:58–69. url: https://www.biodiversitylibrary.org/partpdf/119381.

[34] Baneh H, Hafezian SH. Effect of environmental factor on growth traits in Ghezel sheep. African Journal of Biotechnology. 2009; 8:2903–2907. https://www.ajol.info/index.php/ajb/article/view/60943.

[35] Chriha A, Ghadri G. Caprine in the Arab world. 2. ed. Department of Livestock Production. Fateh University: Libby Conservation of Biodiversity and Environments in the Arab Countries. 2001; 478pp.

[36] Zechner P, Zohman F, Solkner J, Bodo I, Habe F, Marti E, et al. Morphological description of the Lipizzan horse population. Livestock Production Science. 2001; 69:163–177. 10.1016/S0301-6226(00)00254-2.

